# ^99m^Tc-MAA accumulation within tumor in preoperative lung perfusion SPECT/CT associated with occult lymph node metastasis in patients with clinically N0 non-small cell lung cancer

**DOI:** 10.1186/s12885-023-10846-x

**Published:** 2023-04-26

**Authors:** Vanessa Murad, Minseok Suh, Hongyoon Choi, Gi Jeong Cheon, Kwon Joong Na, Young Tae Kim

**Affiliations:** 1 Department of Nuclear Medicine, Seoul National University Hospital, Seoul, Republic of Korea; 2 Department of Nuclear Medicine, Seoul National University College of Medicine, Seoul National University, Seoul, Republic of Korea; 3 Department of Thoracic and Cardiovascular Surgery, Seoul National University Hospital, Seoul, Republic of Korea; 4 Department of Molecular Medicine and Biopharmaceutical Sciences, Graduate School of Convergence Science and Technology, Seoul National University, Seoul, Republic of Korea; 5 Cancer Research Institute, Seoul National University College of Medicine, Seoul National University, Seoul, Republic of Korea; 6 Institute of Radiation Medicine, Medical Research Center, Seoul National University, Seoul, Republic of Korea

**Keywords:** Lung perfusion scintigraphy, Single-photon emission tomography (SPECT) / computed tomography (CT), ^99m^Tc-MAA, Non-small cell lung cancer, Occult nodal metastasis, Imaging biomarker.

## Abstract

**Background:**

^99m^Tc-MAA accumulation within the tumor representing pulmonary arterial perfusion, which is variable and may have a clinical significance. We evaluated the prognostic significance of ^99m^Tc-MAA distribution within the tumor in non-small cell lung cancer (NSCLC) patients in terms of detecting occult nodal metastasis and lymphovascular invasion, as well as predicting the recurrence-free survival (RFS).

**Methods:**

Two hundred thirty-nine NSCLC patients with clinical N0 status who underwent preoperative lung perfusion SPECT/CT were retrospectively evaluated and classified according to the visual grading of ^99m^Tc-MAA accumulation in the tumor. Visual grade was compared with the quantitative parameter, standardized tumor to lung ratio (TLR). The predictive value of ^99m^Tc-MAA accumulation with occult nodal metastasis, lymphovascular invasion, and RFS was assessed.

**Results:**

Eighty-nine (37.2%) patients showed ^99m^Tc-MAA accumulation and 150 (62.8%) patients showed the defect on ^99m^Tc-MAA SPECT/CT. Among the accumulation group, 45 (50.5%) were classified as grade 1, 40 (44.9%) were grade 2, and 4 (4.5%) were grade 3. TLR gradually and significantly increased from grade 0 (0.009 ± 0.005) to grade 1 (0.021 ± 0.005, P < 0.05) and to grade 2–3 (0.033 ± 0.013, P < 0.05). The following factors were significant predictors for occult nodal metastasis in univariate analysis: central location, histology different from adenocarcinoma, tumor size greater than 3 cm representing clinical T2 or higher, and the absence of ^99m^Tc-MAA accumulation within the tumor. Defect in the lung perfusion SPECT/CT remained significant at the multivariate analysis (Odd ratio 3.25, 95%CI [1.24 to 8.48], p = 0.016). With a median follow-up of 31.5 months, the RFS was significantly shorter in the defect group (p = 0.008). Univariate analysis revealed that cell type of non-adenocarcinoma, clinical stage II-III, pathologic stage II-III, age greater than 65 years, and the ^99m^Tc-MAA defect within tumor as significant predictors for shorter RFS. However, only the pathologic stage remained statistically significant, in multivariate analysis.

**Conclusion:**

The absence of ^99m^Tc-MAA accumulation within the tumor in preoperative lung perfusion SPECT/CT represents an independent risk factor for occult nodal metastasis and is relevant as a poor prognostic factor in clinically N0 NSCLC patients. ^99m^Tc-MAA tumor distribution may serve as a new imaging biomarker reflecting tumor vasculatures and perfusion which can be associated with tumor biology and prognosis.

**Supplementary Information:**

The online version contains supplementary material available at 10.1186/s12885-023-10846-x.

## Background

Lung perfusion scan with technetium-99 m-labeled macro-aggregates of albumin (^99m^Tc-MAA) has been used for the evaluation of pulmonary regional perfusion and to predict postoperative lung function [1]. ^99m^Tc-MAA particles are distributed in the pulmonary vasculature in direct proportion to local blood flow, and due to their size (in the range of 10–90 μm), they are trapped on the first pass in arterioles and perialveolar capillaries, which diameter is approximately 25 μm and 10 μm respectively. A cold region in the scan represents decreased or absent pulmonary arterial perfusion in the parenchyma [2–4].

With the advent of single-photon emission tomography (SPECT)/computed tomography (CT), more precise and three-dimensional assessments are now possible [5–8]. Even though the purpose of SPECT/CT is prediction of postoperative lung function, the ^99m^Tc-MAA particles could be variably distributed within the tumor because of a dual vascular blood supply [9–12]. This can be related to the aberrant architecture and dynamics of the tumor vasculature which have distinctive features compared to normal vasculature. Angiogenesis, promoted by different factors produced by both the tumor and its microenvironment, leads to the molding of new blood vessels mainly arising from small vessels or capillaries; but these new vessels are typically immature, tortuous, irregular, and hyperpermeable [13–15]. These characteristics are essential for tumor growth and, as this occurs, vascular remodeling also progresses, representing an essential factor for invasion and metastasis [16]. Of note, as ^99m^Tc-MAA distribution depends on pulmonary arterial perfusion, we have hypothesized that the ^99m^Tc-MAA accumulation within the tumor can be used to evaluate the arteriolar-capillary bed architecture and functional status.

In this study, to find the clinical significance of ^99m^Tc-MAA within the tumor, we assessed the association of ^99m^Tc-MAA accumulation patterns with occult nodal metastasis and lymphovascular invasion, as well as recurrence-free survival (RFS) in clinically N0 non-small cell lung cancer (NSCLC).

## Methods

### Patient enrollment

A total of 298 NSCLC patients who underwent preoperative lung perfusion SPECT/CT and surgery from January 2015 to December 2019 in our institution were retrospectively enrolled. Fifty-nine patients were excluded from the study: 46 patients with clinical nodal stage of N1 or higher, 10 patients with confirmed distant metastases, and 3 patients in which the complete information on the pathology or follow-up was not available. Finally, 239 patients with clinically N0 status were retrospectively evaluated. None of the patients received any treatment prior to the imaging study or surgery. The clinical and pathological TNM classifications were assigned according to the TNM eighth edition [17].

### Image acquisition

Lung perfusion scan and SPECT/CT were acquired using a SPECT/CT scanner (NM/CT670; GE Healthcare, USA) equipped with low-energy high-resolution collimators. Planar scans were obtained 3–5 min after the intravenous administration of ^99m^Tc-MAA with a dose of 185 MBq. Immediately after the planar scan acquisition, SPECT/CT images were acquired. CT images were obtained using the following parameters: tube voltage of 120 kV, tube current of 40 mA with autoMa function, and matrix of 512 × 512. Then, SPECT images were acquired using the following parameters: energy peak of 140.5 KeV with 10% window, step-and-shoot mode acquisition 15 s/frame (16 s/step and 60 steps/detector) with 3° angular increment, and body contour scanning option. Extra-window for scatter correction was set at 120 KeV with a 10% window. SPECT images were reconstructed using an iterative ordered subset expectation maximization (OSEM) algorithm (two iterations and ten subsets) with CT-based attenuation correction, scatter correction and resolution recovery. Reconstructed images were set at the matrix of 128 × 128 with a slice thickness of 3.87 mm and a zoom factor of 1.5.

### Image analysis and ^99m^Tc-MAA distribution classification

**Fig. 1:**
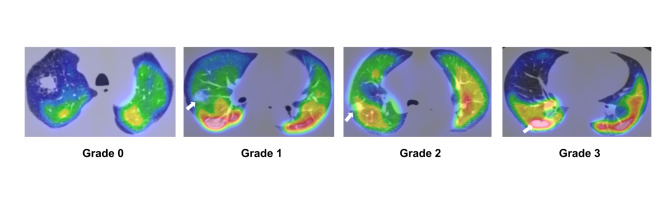
Lung perfusion SPECT/CT visual assessment of ^99m^Tc-MAA distribution within the tumor. The complete absence of ^99m^Tc-MAA in the tumor was considered a defect (0). The presence of ^99m^Tc-MAA particles within the tumor was considered as accumulation, which was correlated with the degree of distribution in the adjacent normal parenchyma: (1) less than that, (2) equal to, and (3) greater than

The images were reviewed on the vendor-supplied software (Volumetric MI™; GE Healthcare). All scans were reviewed separately by two nuclear medicine physicians (VM, MS), masked to the patient medical history. ^99m^Tc-MAA distribution in the tumor was determined based on visual assessment and classified into 4 grades (Fig. 1) after the consensus of both readers. Grade 0 uptake shows complete absence with a defect in the tumor. On the contrary, the presence of ^99m^Tc-MAA particles within the tumor was considered as accumulation and defined as grade 1 when the uptake was less than that of the adjacent parenchyma, grade 2 to be equal to that of the adjacent parenchyma, and grade 3 when greater than that of the adjacent parenchyma. Patients with grade 0 uptake were assigned to the “defect” group, and those with grade 1–3 uptake were classified into the “accumulation” group (Fig. 1).

For the quantitative analysis, standardized tumor to lung ratio (TLR) was defined and calculated as follows:

$$\begin{array}{l}{\rm{TLR}}(\% {\rm{/ml}}) = \\\frac{{Tumorcount\left( {cps} \right)/Tumorvolume\left( {ml} \right)}}{{Totallungcount\left( {cps} \right)}} \times 100\end{array}$$

Using the MIM software (MIM Encore™, MIM Software Inc., Cleveland, OH), lung and tumor were segmented. Total lung was automatically segmented by the MIM software and the count was measured. The region of interest for the tumor was drawn manually based on the CT images on every image slice and interpolated to acquire a single volume of interest (VOI) for the tumor. Tumor count and tumor volume was generated from the VOI.

### Statistical analysis

The differences in the visual grades according to the quantitative parameter were analyzed by using the Anova test and subsequent post hoc analyses. Demographic variables (age, sex and, smoking status), clinical variables (smoking habit, tumor size, and nodal involvement), and pathological variables (histologic type, tumor size, nodal involvement, lymphatic invasion, and vascular invasion) were compared between the groups defined by ^99m^Tc-MAA uptake. Statistical analyses were performed using the MedCalc statistical packages version 14.8 (MedCalc Statistical Software, Mariakerke, Belgium). A chi-squared test and Student’s t-test were used to show group differences. Univariate and multivariate logistic regression analyses were performed to determine the impact in postoperative nodal upstaging. Additionally, the Kaplan–Meier product-limit method was used to estimate survival times, and differences were estimated using a log-rank test. The influence of parameters on recurrence-free survival was analyzed using a Cox regression analysis. A p-value of < 0.05 was considered statistically significant.

## Results

### Comparison of visual grade according to quantitative parameter

**Fig. 2:**
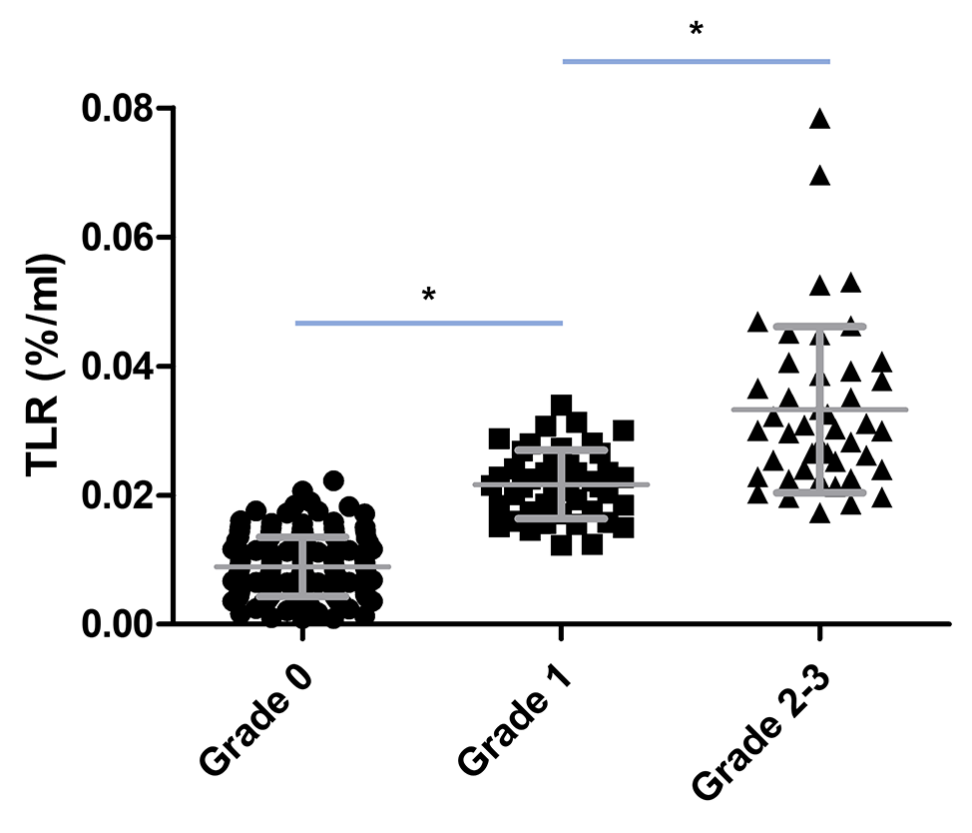
Comparisons of visual grade according to quantitative parameter

Of the 239 patients, 150 (62.8%) were allocated in the defect group and 89 (37.2%) in the accumulation group. Among the accumulation group, 45 (50.5%) were classified as grade 1, 40 (44.9%) were grade 2, and 4 (4.5%) were grade 3. In 4 patients (3 grade 0 and 1 grade 1), TLR could not be obtained due to the absence of the DICOM file, so a comparison of visual grade and quantitative parameters was performed for 235 patients. TLR gradually and significantly increased from grade 0 (0.009 ± 0.005) to grade 1 (0.021 ± 0.005, P < 0.05 compared with grade 0) and to grade 2–3 (0.033 ± 0.013, P < 0.05 compared with grade 2; P < 0.001 with the Anova test; Fig. 2).

### ^99m^Tc-MAA uptake in the tumor associated with clinicopathologic features of NSCLC

The patients’ clinicopathologic characteristics for each group are summarized in Table 1. Compared to the accumulation group, defect group had significantly higher proportion of male gender (69.3% vs. 51.7%, p = 0.007), central location of tumor (44.7% vs. 28.1%, p = 0.011), tumor cell type of non-adenocarcinoma (54.0% vs. 21.4%, p < 0.001), and tumor size bigger than 3 cm (74.7% vs. 48.3%, p < 0.001). No significant differences were observed among adenocarcinoma subtypes. Proportion of clinical stage II & III as well as pathologic stage II & III, was also significantly higher in the defect group (36.6% vs. 10.1%, p < 0.001, and 54.7% vs. 21.4%, p < 0.001, respectively).

**Table 1:** Summary table, ^99m^Tc-MAA uptake in the tumor associated with clinicopathologic features of NSCLC.

Variable	Defect group [n(%)]	Accumulation group [n(%)]	P-value
Total	150 (62.8)	89 (37.2)	
		Grade 1: 39 (43.8)	
		Grade 2: 45 (50.6)	
		Grade 3: 5 (5.6)	
**Age**			n/s
≤ 65 years	45 (30.0)	32 (35.9)	
> 65 years	105 (70.0)	57 (64.1)	
**Sex**			0.007
Female	46 (30.7)	43 (48.3)	
Male	104 (69.3)	46 (51.7)	
**Smoking**			n/s
No	50 (33.3)	25 (28.1)	
Current or previous	100 (66.7)	64 (71.9)	
**Tumor location**			0.011
Central	67 (44.7)	25 (28.1)	
Peripheral	83 (55.3)	64 (71.9)	
**Cell type**			
Adenocarcinoma	69 (46.0)	70 (78.6)	ADC & non-ADC
Lepidic	2	3	< 0.001
Acinar	30	37	
Papillary	14	17	
Micropapillary	2	1	
Solid	11	5	
Adenocarcinoma variants	10	7	
Non-adenocarcinoma	81 (54.0)	19 (21.4)	
**Tumor size**			< 0.001
≤ 3cm	38 (25.3)	46 (51.7)	
> 3cm	112 (74.7)	43 (48.3)	
**Clinical stage**			
I	95 (63.4)	80 (89.9)	I & II, III
T1	38	46	< 0.001
T2a	57	34	
II	47 (31.3)	6 (6.7)	
T2b	22	1	
T3	25	5	
III	8 (5.3)	3 (3.4)	
T4	8	3	
**Pathologic stage**			I & II, III
I	68 (45.3)	70 (78.6)	< 0.001
II	49 (32.7)	12 (13.5)	
III	33 (22.0)	7 (7.9)	
**T stage**			T1 & T2-4
T1	37 (24.7)	43 (48.3)	< 0.001
T2a	55 (36.7)	32 (36.0)	
T2b	21 (14.0)	5 (5.6)	
T3	27 (18.0)	6 (6.7)	
T4	10 (6.7)	3 (3.4)	
**N stage** N0	103 (68.7)	83 (93.3)	N0 & N1-2 < 0.001
N1	27 (18.0)	2 (2.2)	
N2	20 (13.3)	4 (4.5)	

N/S, not significant; ADC, adenocarcinoma

### ^99m^Tc-MAA accumulation associated with occult nodal metastasis

**Fig. 3:**
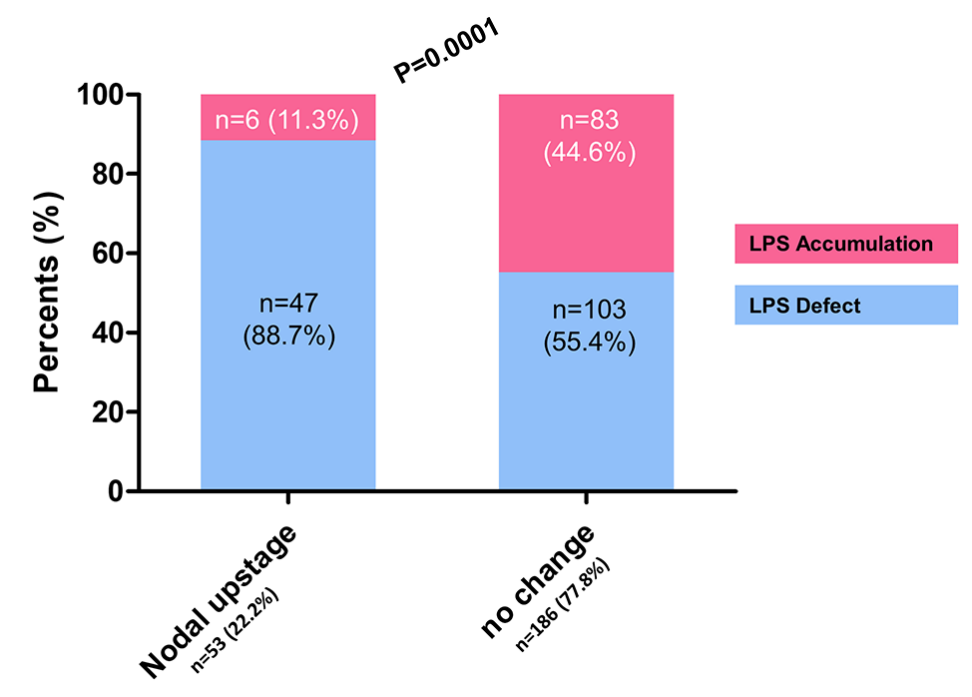
^99m^Tc-MAA accumulation according to nodal upstaging

Occult nodal metastases were found in a total of 53 patients corresponding to 22.2% of the all patients, and the majority of patients with nodal upstage was allocated in the defect group (88.7%) (Fig. 3). TLR was significantly lower in patients with nodal upstage (0.010 ± 0.008 vs. 0.018 ± 0.012, P < 0.001). Univariate analysis identified the following factors as significant predictors for occult nodal metastasis: central location, histology different from adenocarcinoma, tumor size greater than 3 cm representing clinical T2 or higher, and the absence of ^99m^Tc-MAA accumulation within the tumor. Defect in the lung perfusion SPECT/CT remained significant at the multivariate analysis (Odd ratio 3.19, 95%CI [1.22 to 8.34], p = 0.018) (Table 2**).**

**Table 2:** ^99m^Tc-MAA accumulation associated with occult nodal metastasis

Variables	Univariate			Multivariate		
	**Odds ratio**	**95% CI**	**p-value**	**Odds ratio**	**95% CI**	**p-value**
Central location	3.55	1.88 to 6.70	< 0.001	2.77	1.39 to 5.51	0.004
Non-ADC	3.94	2.06 to 7.56	< 0.001	2.26	1.10 to 4.63	0.026
Size (> 3 cm)	5.66	2.30 to 13.89	< 0.001	3.54	1.37 to 9.17	0.009
Defect group	6.31	2.57 to 15.49	< 0.001	3.19	1.22 to 8.34	0.018
Smoking	5.42	2.88 to 10.90	0.123			

ADC, adenocarcinoma

### Prognosis and ^99m^Tc-MAA accumulation

**Fig. 4:**
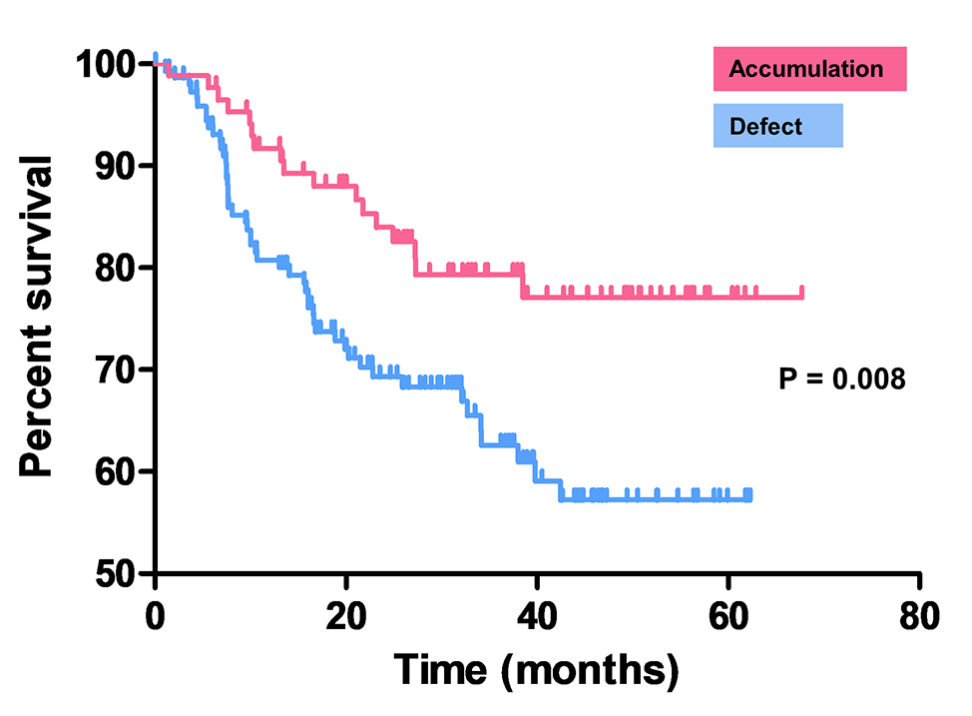
Recurrence-free survival according to ^99m^Tc-MAA distribution

The prognostic significance of ^99m^Tc-MAA distribution within the tumor was evaluated. Compared to the accumulation group, the defect group showed a significantly higher rate of lymphovascular invasion (44.7% vs. 20.2%, p < 0.001) **(Supplementary Fig. 1)**. RFS was evaluated with a median follow-up of 31.5 months. In Fig. 4, Kaplan-Meier curve was shown of RFS stratified by the tumor ^99m^Tc-MAA uptake. The difference between the two groups was significant (p = 0.008). When pairwise comparison was performed by stratifying with visual grade, there was a significant difference between grade 1 and grade 2–3 (P < 0.05), and grade 0 and grade 2–3 (P < 0.01), but no statistically significant difference was observed between grade 0 and grade 1 **(Supplementary Fig. 2)**. Univariate analysis identified the following factors as significant predictors for shorter RFS: cell type of non-adenocarcinoma, clinical stage II-III, pathologic stage II-III, age greater than 65 years, and the absence of ^99m^Tc-MAA accumulation within the tumor. In multivariate analysis, only the pathologic stage remained statistically significant **(**Table 3**)**.

**Table 3:** Prognosis and ^99m^Tc-MAA accumulation

Variables	Univariate			Multivariate		
	**Odds ratio**	**95% CI**	**p-value**	**Odds ratio**	**95% CI**	**p-value**
Non-ADC	2.04	1.26 to 3.31	0.004	1.30	0.76 to 2.20	0.339
cStage II & III	2.37	1.45 to 3.86	0.001	1.33	0.75 to 2.36	0.334
pStage II & III	2.63	1.61 to 4.29	< 0.001	1.91	1.06 to 3.42	0.032
Defect group	2.08	1.20 to 3.61	0.006	1.41	0.78 to 2.56	0.251
Age (> 65)	1.72	0.99 to 3.03	0.048	1.63	0.92 to 2.89	0.094
Central location	1.47	0.91 to 2.40	0.121			
Smoking	1.34	0.78 to 2.30	0.286			

ADC, adenocarcinoma

## Discussion

We evaluated the clinical significance of ^99m^Tc-MAA particles distribution within lung tumors, finding that the absence of ^99m^Tc-MAA accumulation represents an independent risk factor for the presence of occult nodal metastases and is relevant as a poor prognostic factor in NSCLC patients.^99m^Tc-MAA accumulation could be used as a noninvasive imaging method to assess biological factors of NSCLC in terms of tumor vasculature.

Through this study, we confirmed that the distribution of ^99m^Tc-MAA particles within the tumor was variable. This can be largely explained because in initial stages of tumor proliferation, when the vascular architecture and dynamics are not altered, the perfusion within the tumor will remain almost the same as the perfusion of the adjacent parenchyma. On the contrary, when the tumor progresses and/or increases in size, vascular remodeling with disruption of the normal capillary bed and increased resistance due to irregular and tortuous vessels leads to impaired blood flow, which in the images would manifest as a cold region. However, in addition to the hypothesis of vascular remodeling associated with ^99m^Tc-MAA distribution, various clinicopathologic factors may influence the intratumoral uptake of ^99m^Tc-MAA particles.

Firstly, the insufficient blood supply from the pulmonary artery may decrease MAA particle uptake. It is known that lung cancer has a dual blood supply both originating from the pulmonary and the bronchial arteries [18]. The blood flow that supplies the tumor arises in greater proportion from the bronchial arteries, but it has been reported to be related to various factors [9–12]. Previous studies showed that the proportion of dual perfusion was dependent on the location of the tumor, pointing that centrally located tumors have a significantly lower supply from the pulmonary artery [9, 10]. Nakano et al. also reported that tumor size had a negative correlation with the proportion of pulmonary perfusion [10]. In line with the previous studies, we found larger and centrally located tumors at a significantly higher rate in the defect group than in the accumulation group. However, some studies showed partly different results. Nguyen-Kim et al. reported that the dual blood supply of non-small cell lung cancer depends on tumor size and the subtype of the NSCLC [11]. They concluded that tumor with larger volume (> 3.5 cm^3^) and histological type of squamous-cell carcinoma showed significantly higher pulmonary blood supply compared with smaller tumors and adenocarcinoma, respectively. In our study, non-adenocarcinoma including squamous cell carcinoma was observed at a larger proportion in the defect group. These different results could be related to the characteristics of ^99m^Tc-MAA. ^99m^Tc-MAA distribution may not be solely explained by pulmonary supply due to collaterals and abnormal vasculatures in lung tumors. Therefore, tumors with high vascularity identified on contrast CT could be different from ^99m^Tc-MAA accumulation. Further studies using ^99m^Tc-MAA SPECT/CT and perfusion CT for evaluating dual supply of lung tumors may clarify this controversy.

Another factor of ^99m^Tc-MAA uptake in the tumor could be the size of tumor, particularly considering spill-over from surrounding normal lung parenchymal ^99m^Tc-MAA distribution. In our study as well, the spill-over effect cannot be neglected considering that the ratio of small tumors (≤ 3 cm) in the accumulation group was higher and vice versa in the defect group. However, no significant correlation was observed between visual assessment grade and tumor size in the subgroup analysis of the accumulation group (**Supplementary Fig. 3**). Lastly, the major histologic subtype may influence ^99m^Tc-MAA tumor uptake, as it reflects the structural characteristics of cancer [19]. From the subgroup analysis of patients with adenocarcinoma, we observed that the proportion of solid type was higher in the defect group, whereas a higher proportion of lepidic and acinar types were observed in the accumulation group, but fail to meet the statistical significance (Table 1). Overall, the distribution of ^99m^Tc-MAA distribution within the tumor is relevant to various factors, but there is a limit to a causal interpretation with a single factor, thus, it is necessary to elucidate the mechanism through future study.

We showed that the absence of ^99m^Tc-MAA accumulation was associated with the central location of the tumor, the tumor cell type of the non-adenocarcinoma, and the larger tumor size. These factors are known to be associated with a poor prognosis of lung cancer. Correspondingly, in this study, the relevance between occult nodal metastasis, lymphovascular invasion, and RFS and MAA tumor uptake was observed. The presence of lymph node metastasis is crucial in determining the type and extent of lung cancer surgery. Multiple studies have documented a non-negligible rate of pathological N1 or N2 disease in patients with clinical N0 disease, ranging from 15 to 26%, which is known as “nodal upstaging” or “occult nodal metastases” [20–22]. Multiple predictive factors for occult nodal metastasis have been studied, since it has a negative impact on survival, and the most relevant with statistical significance include: male sex, tumor size, tumor grade, histology, and centrally located tumor [22]. We observed consistent results, that large tumor size, tumor cell type of non-adenocarcinoma, and central location of tumor were independent factors in predicting occult nodal metastasis. Though relevance with the above-mentioned factors exists, the absence of ^99m^Tc-MAA accumulation in the tumor remained as an independent predictor for occult nodal metastases. A plausible explanation of the association of ^99m^Tc-MAA and nodal metastasis is abnormal vasculature affecting ^99m^Tc-MAA accumulation patterns. In particular, angiogenesis is accompanied by the lymphangiogenesis process, promoted by proteins such as VEGF-C and VEGF-D, which leads to the development of large and tortuous lymphatic vessels that also facilitates metastatic spread [23]. This may explain why the absence of tracer accumulation in the tumor behaves as an independent risk factor for the presence of occult nodal metastasis and also shorter RFS. Further mechanism studies related to lymphangiogenesis and ^99m^Tc-MAA accumulation are needed to support this hypothesis. Nonetheless, this is the first study to observe the diversity of ^99m^Tc-MAA accumulation within the tumors and to evaluate its clinical significance.

## Conclusion

Our findings demonstrate that ^99m^Tc-MAA particles distribution in preoperative lung perfusion SPECT/CT is an important finding and that the absence of accumulation or defect, represents an independent risk factor for occult nodal metastases, lymphovascular invasion, and shorter RFS. ^99m^Tc-MAA distribution may then serve as an imaging biomarker, representing the arteriolar-capillary bed architecture and functional status of the tumor, for determining the extent of surgery and adjuvant treatment strategy in clinically N0 NSCLC patients.

## Electronic supplementary material

Below is the link to the electronic supplementary material.

Supplementary Material 1

Supplementary Material 2

## Data Availability

All data generated or analysed during this study are included in this published article [and its supplementary information files].
