# Supplementary material for: 99mTc-MAA accumulation within tumor in preoperative lung perfusion SPECT/CT associated with occult lymph node metastasis in patients with clinically N0 non-small cell lung cancer
Source: BMC Cancer. 2023 Apr 26;23:381. doi: 10.1186/s12885-023-10846-x (PMC10131419; doi:10.1186/s12885-023-10846-x)
Supplement: Supplementary file 1 — Supplementary Material 1 [file 12885_2023_10846_MOESM1_ESM.docx]

**Supplementary Data**

Clinical significance of ^99m^Tc-MAA distribution within tumor in preoperative lung perfusion SPECT/CT for evaluation of patients with clinically N0 non-small cell lung cancer

**Supplementary Figure 1.** ^99m^Tc-MAA accumulation according to lymphovascular invasion.

**
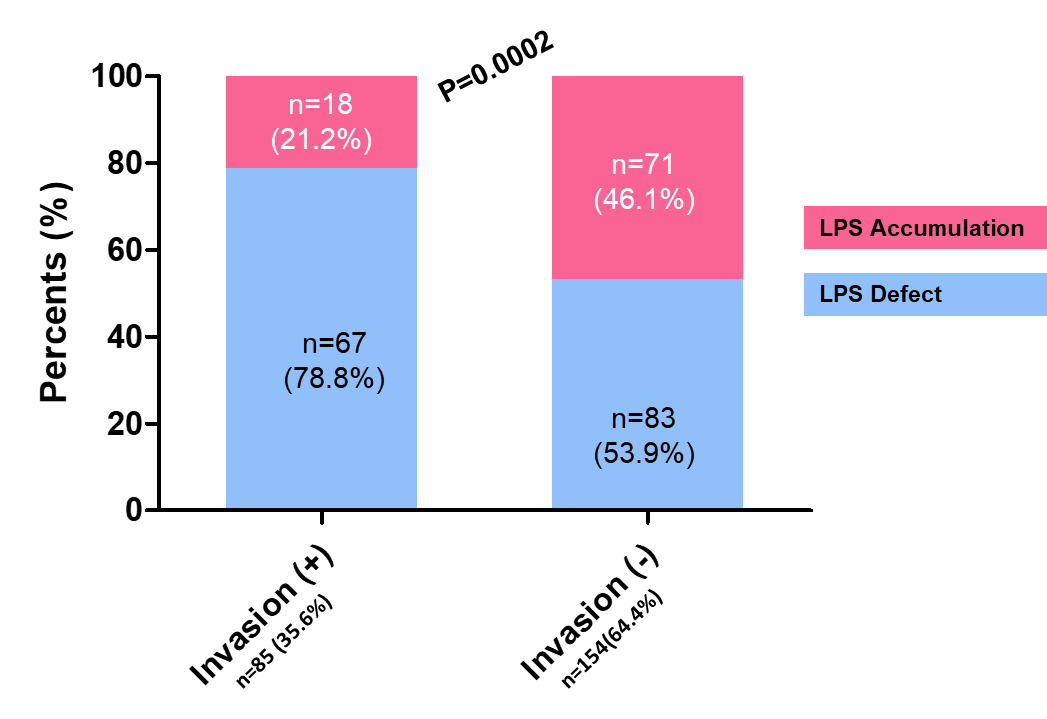
**

.

**Supplementary Figure 2.** Recurrence-free survival according to visual grade.

**
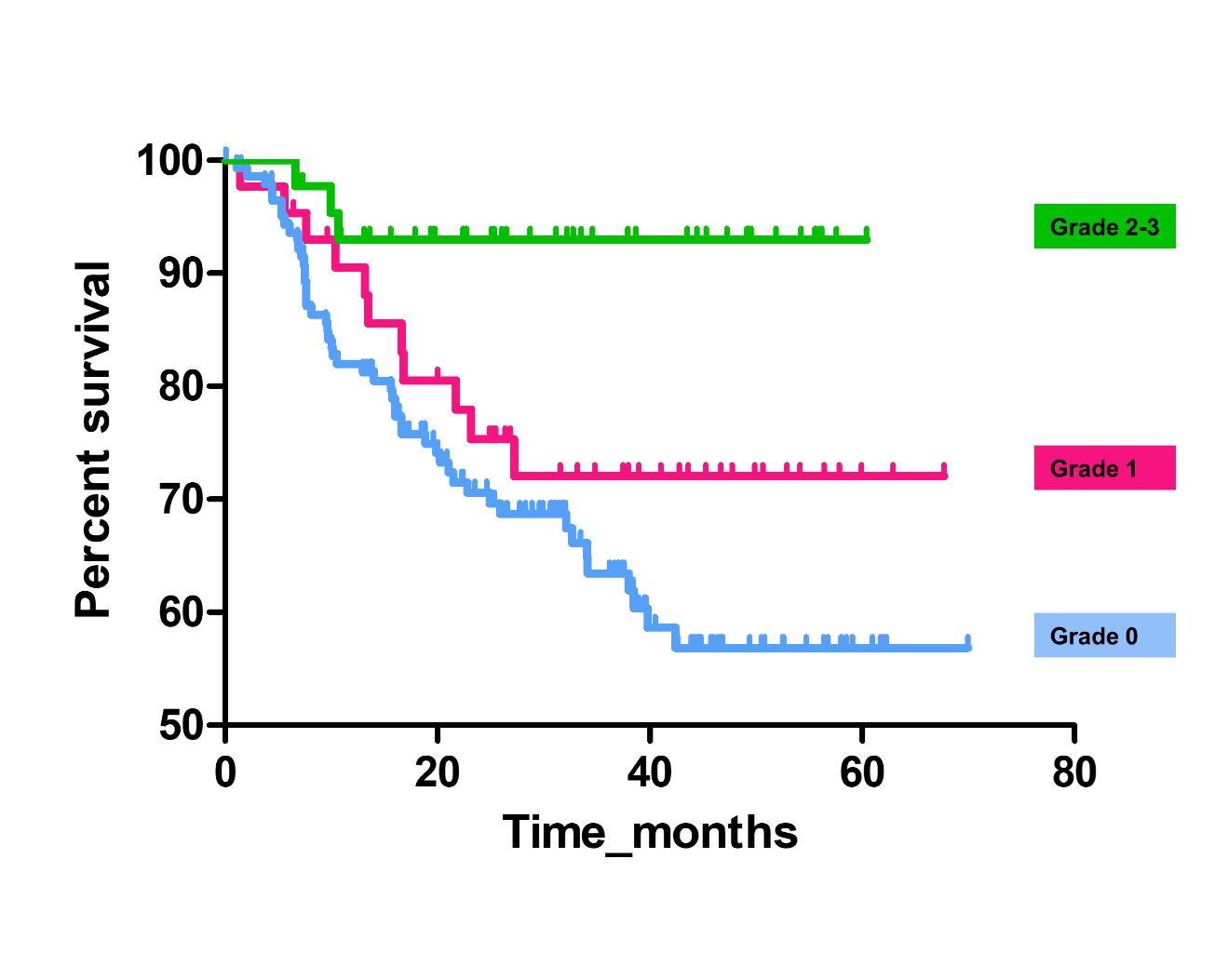
**

**Supplementary Figure 3.** Relationship between tumor size and visual grade.
